# Fast exchange of strontium between hair and ambient water: Implication for isotopic analysis in provenance and forensic studies

**DOI:** 10.1371/journal.pone.0233712

**Published:** 2020-05-29

**Authors:** Lihai Hu, Diego P. Fernandez, Thure E. Cerling, Brett J. Tipple

**Affiliations:** 1 Department of Geology and Geophysics, University of Utah, Salt Lake City, UT, United States of America; 2 Department of Biology, University of Utah, Salt Lake City, UT, United States of America; 3 Global Change and Sustainability Center, University of Utah, Salt Lake City, UT, United States of America; University of South Florida, UNITED STATES

## Abstract

Trace elements in hair originate from intake (e.g., diet, inhalation, skin absorption), are transported in the bloodstream, and then incorporated during hair formation. However, the trace element abundance and isotopic compositions may be altered by post-eruption environmental processes. Such alterations must be addressed to obtain a meaningful interpretation of hair analysis for biomonitoring. In this study, we used strontium (Sr) isotopic analysis together with sorption kinetics of ionic Sr to quantify the rate and extent of replacement of endogenous Sr in hair by exogenous Sr from ambient water. We found that with only 10 minutes of exposure at room temperature (22°C), more than 30% of original endogenous Sr in hair was replaced with exogenous Sr from the solution. After 16 days of exposure to the solution, more than 90% of endogenous Sr was replaced, with a warmer temperature (60°C) accelerating the exchange substantially. We also found that acid leaching of exposed hair did not remove or isolate the exogenous Sr; therefore, neither the original endogenous nor the exogenous ^87^Sr/^86^Sr signal could be separated. Nonetheless, these findings illustrated that the quantitative correlation between the fraction of exogenous Sr and the soaking time, if established, could be used to estimate the length of water contact time for hair in forensic studies. Even if such time since initial contact cannot be established, the combination of acid leaching and ^87^Sr/^86^Sr analysis of hair samples may still be valuable in provenance studies to identify recent changes in the exogenous Sr pool, including movements or changes in water source.

## Introduction

Isotopic variation of strontium (Sr) in plant and animal tissue relates to weathering of local rocks, exposure to surface and groundwaters, and atmospheric aerosol deposition. Studies on such variation provide opportunities to identify local versus non-local individuals [1], habitat use [2], natal origins of migratory species [3,4], etc. Hair has been used as a biomonitor of trace element exposures such as Hg [5], and Sr isotopes in hair have shown promise for provenance, forensic, and archaeological studies [6–10]. Therefore, understanding the sources of Sr in hair is crucial for the elucidation of environmental or forensic hypotheses.

Strontium from the weathering of bedrock is incorporated into local soil [11] and assimilated by plants [12], which are consumed by animals and humans. The daily ingestion of Sr for an average person is 1.9‒3.3 mg [13,14]. While most ingested Sr is either lost through excretion and perspiration or retained in bone, a small fraction (~0.2 ng/day) is incorporated into the bloodstream and can be integrated into hair [13]. As Sr isotopes are not fractionated in biological cycles, the radiogenic Sr isotopic signature (reported as the isotopic ratio ^87^Sr/^86^Sr) in pristine hair is thus a record of the ^87^Sr/^86^Sr ratio of blood, which can be traced back to local geology. Moreover, the longitudinal variation of ^87^Sr/^86^Sr in hair was correlated with recent travel events for modern humans [6] and modern horse [1], due to the changes of environmental sources with distinct ^87^Sr/^86^Sr ratios. This raises the potential of using such variation to identify travel events of forensic and archaeological samples with unknown histories, and even to identify their provenance [7–10]. On the other hand, several studies have found that untreated hair, acid-leached hair, and leachate differed in their ^87^Sr/^86^Sr ratios [6,15], indicating the existence of exogenous Sr incorporated in hair. One study on pig tissues found that ^87^Sr/^86^Sr ratios in bristle samples, both before and after acid leaching, were distinctly different from the ^87^Sr/^86^Sr ratios of other internal tissues, indicating the existence of exogenous Sr in the bristle [16]. Multiple Sr sources, therefore, complicate the interpretation of Sr isotopic analysis for keratinized tissues.

It has been long known that hair behaves like an ion-exchange resin which sorbs and desorbs dissolved metal ions, including Sr, in aqueous solution depending on pH [17,18]. One previous study [19] found that elephant hair contained more than ten times Sr contamination than giraffe hair, explained by the fact that elephants wallow while giraffes do not. Given most humans bathe regularly, it is likely their hair incorporates exogenous Sr contamination from ambient water. For a recently traveled individual, the Sr isotopic signature of the new location would pass through diet and blood, and eventually be incorporated as the endogenous Sr in the new grown hair segment. At the same time, such a signature would also likely be added into the older part of the hair as exogenous Sr through surface contamination. It is thus essential to understand the rate and extent of incorporation of exogenous Sr when using ^87^Sr/^86^Sr ratios of hair for provenance and forensic studies.

Here we quantified the rate and extent of the incorporation of exogenous Sr into human hair through sorption experiments. We exposed hair subsamples to river water with a distinct ^87^Sr/^86^Sr ratio for different time lengths at two temperatures and studied the effect of replacing the water and dry-wet cycles. The rate and extent of the incorporation of exogenous Sr were quantified using a simple binary mixing model. In addition, acid leaching had been found to be more efficient in removing Sr from hair subsamples than other washing methods using the reagents of water, chloroform-methanol mixture, or IAEA procedure [15]. Therefore, we also tested whether acid leaching could recover the two Sr sources from the soaked hair subsamples: the original hair Sr and the exogenous Sr incorporated from the water during soaking. Finally, we evaluated the use of Sr isotopic analysis of hair in provenance and forensic studies with the consideration of exogenous contamination.

## Materials and methods

### Samples and reagents

One ponytail of human hair (cut from the middle of the original hair) collected with no identifiable information in a barber’s shop in Salt Lake City, Utah, USA. This research does not need IRB review because there is no private information involved, which has been confirmed by the IRB board of the University of Utah. The length of the collected sample was about 15 cm. The color of the ponytail was brown and black. The two ends of the ponytail were cut into ~2 cm long segments: the segments of the distal end were grouped together and called “tip hair,” while the segments of the proximal end were called “mid hair.”

The river water used in this experiment was collected from Bear River near Corinne, UT, on March 18, 2018, using a clean 9.5-liter low-density polyethylene (LDPE) container. The Bear River where the water sample was collected is owned by the state of Utah and was not in a state or national park. No permission was needed for water sample collection as the sample was taken from a public access point. The water was then filtered through hydrophilic polytetrafluoroethylene (PTFE) 0.45 μm, 25mm cartridge filters in the lab. The pH of both the unfiltered and the filtered water was measured to be 8.

The ultrapure water used in this study was obtained from a Milli-Q® Academic A10 system (MilliporeSigma, Burlington, MA, USA) with a resistivity >18 MΩ. The concentrated nitric acid (HNO_3_) used in this study was ultrapure ARISTAR® ULTRA nitric acid (67–70%), manufactured by BDH Chemical. The concentrated hydrochloric acid (HCl) used in this study, unless otherwise stated, was ultrapure PlasmaPure Plus hydrochloric acid (32–35%), manufactured by SCP SCIENCE. The concentrated hydrogen peroxide (H_2_O_2_) used in this study was ULREX® II Ultrapure 30% hydrogen peroxide, manufactured by J.T. Baker.

### Experiment designs

Several ~100 mg subsamples of the mid hair and the tip hair were used in this experiment. Each one was dipped in 40 mL of the filtered Bear River water for the time and conditions listed in Table 1. Six sets of subsamples of each mid and tip hair, were continuously soaked in Bear River water at 22°C (room temperature) for various times (label: “10 min”, “1 hr”, “6 hr”, “1 d”, “2 d”, and “16 d”). One set of subsamples (label: “Refresh (16 d)”) was put into Bear River water for four days at 22°C until the water was replaced, and the process repeated four times. One set of subsamples (label: “Wet-dry (16 d)”) was put into Bear River water for two days and then dried for 2 days in a laminar flow bench at 22°C, repeated four times. One set of subsamples (label: “60°C (2 d)”) was continuously soaked for 2 days in Bear River water at 60°C to assess the influence of temperature. Two unsoaked subsamples (“Original-1” and “Original-2”) of each mid and tip hair were used as control groups.

**Table 1:** Setting for soaking of human hair in river water.

Label ID	Procedure of soaking
Original-1	Intact hair, no soaking
Original-2	Intact hair, no soaking
10 min	Continuous soaking for 10 minutes in 40 mL water at room temperature (22°C)
1 hr	Continuous soaking for 1 hour in 40 mL water at room temperature
6 hr	Continuous soaking for 6 hours in 40 mL water at room temperature
1 d	Continuous soaking for 1 day in 40 mL water at room temperature
2 d	Continuous soaking for 2 days in 40 mL water at room temperature
16 d	Continuous soaking for 16 days in 40 mL water at room temperature
Refresh (16 d)	Soaking for 4 days in 10 mL water, repeat 4 times with fresh water at room temperature
Wet-dry (16 d)	Soaking for 2 days in 10 mL water, drying for 2 days in a laminar flow bench, repeat 4 times with fresh water at room temperature
60°C (2 d)	Continuous soaking for 2 days in 40 mL water in an oven at 60°C

After the soaking procedure, each subsample was rinsed with Milli-Q water, dried in a laminar flow bench and split into two fractions. One fraction (approximately one-third of each subsample) was later digested. The other fraction (approximately two thirds) was leached with 3–5 mL 0.1 M HCl in an acid-leached centrifuge tube in an ultrasonic bath for 10 minutes, repeated three times, following Treatment 3 in Tipple et al. [15]. All three solutions from the leaching procedure were decanted and combined into another acid-leached centrifuge tube labeled as “leachate.” The leached residue of each hair subsample, called “leached hair,” was rinsed with Milli-Q water and dried in a laminar flow bench. The unsoaked subsamples (“Original-1” and “Original-2”) were processed through the same acid leaching procedure as other soaked subsamples. All dried hair subsamples were then digested using a microwave digestion system. Sr concentrations and ^87^Sr/^86^Sr ratios of all hair subsamples and leachates were measured and the results are reported in Table 4. Sr concentration and ^87^Sr/^86^Sr ratio were also measured for the filtered Bear River water, unsoaked mid hair (*n* = 6), and unsoaked tip hair (*n* = 7) (results reported in Table 3).

### Microwave digestion of hair samples

All hair samples were digested in 6 mL PTFE vessels with a mixture of 2 mL concentrated HNO_3_ and 0.25 mL concentrated H_2_O_2_, using Milestone ETHOS EZ microwave acid-assistant digestion system (Milestone, Inc., Shelton, CT, USA) following the method described in Tipple et al. [15]. The digestion system was heated to 200°C at a rate of 13.3°C/min and then left at 200°C for 15 minutes. After cooling down, a 100 μL aliquot of the total ∼2 mL digest from each sample was transferred to a 15 mL tube and brought up to 10 mL with Milli-Q water for Sr concentration analysis. All dilutions were done gravimetrically. The remaining solutions were transferred into 7 mL acid-leached perfluoroalkoxy alkanes (PFA) round-interior vials (Savillex Corporation, Minnetonka, MN, USA) for Sr purification and isotopic analysis.

### Sr concentration analysis

The Sr concentrations of all samples were measured using inductively coupled plasma quadrupole mass spectrometry (ICP-Q-MS) (Agilent 7500ce; Agilent Technologies, Inc., Santa Clara, CA, USA). A quartz dual cyclonic spray chamber with a PFA nebulizer, a quartz torch with a sapphire injector, and platinum-tipped cones were used. Indium was added to all samples as an internal standard at the concentration of 50 μg*/*kg. The certified reference material No. 13 human hair (National Institute for Environmental Studies, Japan) was digested using the microwave digestion system and measured together with SRM^®^ 1643e (NIST, Gaithersburg, MD, USA). The measured strontium concentration of the SRM^®^ 1643e standard through the life cycle of the study was 334 ± 33 ng/g (2SD, *n* = 15), which is consistent with the certified value of 323.1 ± 3.6 ng/g. Although there is no certified value for the Sr concentration of the No. 13 hair standard, its Sr concentration was measured in this study as 2.7 μg/g, which is within the range of 2.3 ± 0.7 μg/g (2SD, *n* = 8) from Tipple et al. [15], and its measured concentrations of Cu (15.3 μg/g) and Sb (40 ng/g) were within the ranges of their certified values (15.3 ± 1.3 μg/g and 42 ± 8 ng/g, respectively).

### Sr purification and isotopic analysis

Hair digests in concentrated HNO_3_ from the microwave digestion were dried down at 200°C and re-dissolved with 1 mL 2 M HNO_3_ in 7 mL PFA vials. River water samples were also dried down at 200°C and re-dissolved with 1 mL 2 M HNO_3_ in 7 mL PFA vials. Those solutions were loaded into the prepFAST MC™ automatic sample purification system (Elemental Scientific, Omaha, Nebraska). The CF-MC-SrCa-1000 column with 1000 μL resin (Elemental Scientific, Omaha, Nebraska) was used. The chromatographic procedure is listed in Table 2. Acid blank, SRM^®^ 987 Sr isotopic standard (National Institute of Standards and Technology, Gaithersburg, MD, USA), and an in-lab carbonate standard, all dissolved in 1 mL 2 M HNO_3_, were processed together with samples using the same procedure. The eluates in 6 M HNO_3_ were dried at 180°C and re-dissolved with 2.4% v/v HNO_3_ in 12 mL acid-leached PFA tubes (PFA-T16; Elemental Scientific, Omaha, NE, USA) for isotopic analysis.

**Table 2:** Chromatographic procedure of Sr purification method.

Step	Reagent	Volume (μL)	Flow rate (μL/min)
Condition column	2 M HNO_3_	5000	2000
Load sample	Sample in 2 M HNO_3_	1000	1000
Wash matrix	2 M HNO_3_	3000	2000
Elute Sr	6 M HNO_3_	5000	1000
Elute Ca	0.1 M HCl	5000	1000
Clean column	0.1 M HCl	1000	5000

Strontium isotopic analyses were performed at the Strontium Isotope Geochemistry Laboratory in the Department of Geology and Geophysics at the University of Utah using a Thermo Scientific™ Neptune Plus™ high-resolution multi-collector inductively coupled plasma mass spectrometer (MC-ICP-MS; Thermo Fisher Scientific, Bremen, Germany). Sample solutions in 2.4% v/v HNO_3_ were aspirated through a 100 μL/min autosampler probe into the MC-ICP-MS using a PFA nebulizer, a double-pass quartz spray chamber, quartz torch, and nickel sample and skimmer cones. Isotopes ^82^Kr, ^83^Kr, ^84^Sr, ^85^Rb, ^86^Sr, ^87^Sr, and ^88^Sr were simultaneously measured in L4, L3, L2, L1, C, H1, and H2 Faraday cups, respectively. Measurements of samples were made using a static multi-collector routine that consisted of one block of 72 cycles with an integration time of 4.194 s/cycle. Before every sample measurement, 36 cycles of 2.4% v/v HNO_3_ solution were measured for blank-correction. ^84^Sr and ^86^Sr have isobaric interferences from ^84^Kr and ^86^Kr, respectively. ^87^Sr has an isobaric interference from ^87^Rb. The interferences of ^84^Sr and ^86^Sr were corrected by subtracting the amount of ^84^Kr and ^86^Kr corresponding to the ^83^Kr signal. The interference of ^87^Sr was corrected by subtracting the amount of ^87^Rb corresponding to the ^85^Rb signal. The ^85^Rb/^88^Sr ratios of all samples were between 1×10^−5^ to 8×10^−5^. Instrumental mass fractionation was corrected by normalizing ^86^Sr/^88^Sr to 0.1194 [20] using the exponential law. An iterative procedure for interference and mass bias correction was applied until convergence. Strontium isotopic compositions are reported as ^87^Sr/^86^Sr ratios. A solution of the SRM^®^ 987 standard of 100 ng/g, with a certified ^87^Sr/^86^Sr ratio of 0.71034 ± 0.00026, was analyzed after every three samples to verify measurement accuracy. The measured ^87^Sr/^86^Sr ratio of the SRM^®^ 987 standard through the life cycle of the study (two analytical sessions in two separate days) was 0.710295 ± 0.000025 (2SD, *n* = 37). The ^88^Sr signal intensity ranges of the SRM^®^ 987 standard and samples were 3.7–6.6 volts (V) (4.8 ± 1.5, 2SD, *n* = 37) and 1.3–5.9 V (3.2 ± 1.8, 2SD, *n* = 66), respectively. The SRM^®^ 987 standard of 10 ng/g was run in another analytical session to check the uncertainty of ^87^Sr/^86^Sr analysis with low signal intensity. We obtained ^87^Sr/^86^Sr ratio of 0.71032 ± 0.00010 (2SD, *n* = 16) with the ^88^Sr signal intensity of 0.53 ± 0.01 V (2SD, *n* = 16).

## Results and discussion

### Original Sr concentrations and ^87^Sr/^86^Sr ratio of the unsoaked hair and the water

Six replicates of the unsoaked mid hair and seven replicates of the unsoaked tip hair were analyzed to obtain the original Sr concentration ([Sr]) and ^87^Sr/^86^Sr ratio (Table 3). The [Sr] in unsoaked mid hair was 13.3 ± 5.7 μg/g (2SD, *n* = 6) and its ^87^Sr/^86^Sr ratio was 0.70909 ± 0.00010 (2SD, *n* = 6). The [Sr] in unsoaked tip hair was 15.9 ± 2.0 μg/g (2SD, *n* = 7) and its ^87^Sr/^86^Sr was 0.70901 ± 0.00004 (2SD, *n* = 7). The Sr concentration in the filtered Bear River water was measured to be 460 μg/L, while the ^87^Sr/^86^Sr ratio was measured to be 0.71408.

**Table 3:** Original unsoaked hair composition.

Sample	Weight (g)	[Sr] (μg/g)	^87^Sr/^86^Sr
Mid 1	0.0672	13.6	0.70907
Mid 2	0.0756	12.1	0.70910
Mid 3	0.1195	11.0	0.70906
Mid 4	0.0584	10.1	0.70918
Mid 5	0.0295	15.4	0.70906
Mid 6	0.0320	17.7	0.70906
**Mid Average**		**13.3**	**0.70909**
**2SD**		**5.7**	**0.00010**
Tip 1	0.0385	15.3	0.70900
Tip 2	0.0559	15.5	0.70899
Tip 3	0.0603	15.3	0.70898
Tip 4	0.0460	15.9	0.70901
Tip 5	0.0467	14.9	0.70900
Tip 6	0.0297	17.6	0.70905
Tip 7	0.0466	17.1	0.70901
**Tip Average**		**15.9**	**0.70901**
**2SD**		**2.0**	**0.00004**

### Fast exchange of Sr between hair and river water

**Fig 1:**
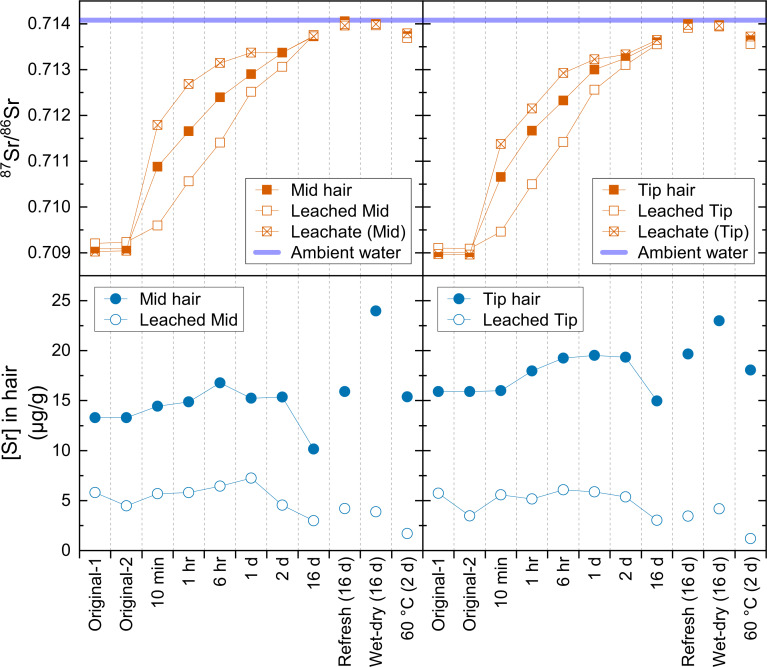
Soaking of human hair in river water and leaching of Sr from the hair.

The results of the soaking in river water and leaching in diluted HCl of the hair subsamples are reported in Table 4. ^87^Sr/^86^Sr and [Sr] of the soaked hair subsamples, their leached residues, and the leachates with respect to their soaking time in the river water are shown in Fig 1.

**Table 4:** Sorption and leaching of Sr in human hair.

Sample ID	Total hair weight (mg)	Soaked hair			Leached hair				Leachate				Δ^87^Sr/^86^Sr (leached–leachate)
		[Sr] (μg/g)	^87^Sr/^86^Sr	*f* ** (%)	[Sr] (μg/g)	Weight (mg)	^87^Sr/^86^Sr	*f* ** (%)	[Sr] (ng/g)	Weight (g)	^87^Sr/^86^Sr	*f* ** (%)	
**Mid hair**													
Original-1		13.3*	0.70909*		5.8	75.5	0.70920		69.8	13.9	0.70902		0.00018
Original-2		13.3*	0.70909*		4.5	58.6	0.70923		34.2	12.9	0.70904		0.00019
10 min	98.2	14.4	0.71088	35.9	5.7	44.2	0.70960	10.2	32.7	12.2	0.71179	54.1	0.00219
1 hr	110.7	14.9	0.71165	51.3	5.8	58.2	0.71056	29.5	41.7	11.9	0.71268	71.9	0.00212
6 hr	105.1	16.8	0.71240	66.3	6.4	51.5	0.71141	46.5	26.9	13.9	0.71315	81.4	0.00174
1 d	111.8	15.2	0.71290	76.4	7.2	53.5	0.71251	68.5	28.6	12.8	0.71337	85.8	0.00086
2 d	106.6	15.4	0.71337	85.8	4.5	44.1	0.71306	79.6	41.1	13.6	0.71337	85.8	0.00031
16 d	106.4	10.2	0.71374	93.2	3.0	48.7	0.71376	93.6	39.1	12.5	0.71373	93.0	0.00003
Refresh (16 d)	103.3	15.9	0.71406	99.6	4.2	57.3	0.71399	98.2	51.4	14.6	0.71396	97.6	0.00003
Wet-dry cycle (16 d)	103.3	24.0	0.71399	98.2	3.9	45.9	0.71397	97.8	62.6	12.6	0.71400	98.4	0.00003
60°C (2 d)	109.2	15.4	0.71373	93.0	1.7	50.3	0.71369	92.2	66.6	14.0	0.71379	94.2	0.00010
**Tip hair**													
Original-1		15.9*	0.70901*		5.7	43.7	0.70910		23.7	14.0	0.70897		0.00013
Original-2		15.9*	0.70901*		3.5	116.9	0.70909		81.1	12.3	0.70896		0.00013
10 min	98.7	16.0	0.71066	32.5	5.6	51.5	0.70946	8.9	33.3	12.4	0.71137	45.7	0.00191
1 hr	97.1	18.0	0.71166	52.3	5.2	54.2	0.71049	29.2	33.5	14.5	0.71215	61.3	0.00166
6 hr	104.7	19.3	0.71232	65.3	6.1	41.6	0.71142	47.5	35.9	12.3	0.71292	76.8	0.00150
1 d	99.4	19.5	0.71300	78.7	5.9	53.6	0.71256	70.0	34.9	11.9	0.71323	83.0	0.00067
2 d	101.6	19.3	0.71325	83.6	5.4	41.8	0.71310	80.7	29.2	13.3	0.71333	85.0	0.00023
16 d	105.3	15.0	0.71359	90.3	3.0	46.7	0.71355	89.5	24.2	12.1	0.71364	91.2	0.00009
Refresh (16 d)	106.0	19.7	0.71399	98.2	3.4	54.3	0.71391	96.6	39.6	13.8	0.71397	97.8	0.00006
Wet-dry cycle (16 d)	93.2	23.0	0.71397	97.8	4.2	49.1	0.71394	97.2	62.3	13.5	0.71396	97.6	0.00002
60°C (2 d)	99.4	18.1	0.71364	91.3	1.2	59.1	0.71356	89.7	52.2	13.2	0.71372	92.8	0.00016

* Average values of original hair from Table 3.

** *f* is "fraction of exogenous Sr."

The [Sr] in both mid and tip soaked hair subsamples (solid circles in Fig 1) did not exhibit much variation except for the sets of “16 d” and “Wet-dry (16 d)” subsamples. The [Sr] of “16 d” subsamples were slightly lower than other soaked subsamples, while the [Sr] of “Wet-dry (16 d)” subsamples were higher than others. Previous studies have found the concentrations of trace elements, including Sr, increase from the proximal end of the hair to the distal end [e.g., 6,21], indicating the accumulation of trace element contaminations in the hair over time. Human hair in contact with water frequently during bathing is analogous to the wet-dry cycles in this study. Therefore, the longitudinal increase of the [Sr] observed previously [e.g., 6,21] is consistent with the higher [Sr] of the “Wet-dry (16 d)” subsamples shown in Fig 1. Possibly, the lower [Sr] in “16 d” subsamples may be due to the change of hair structure with such an extended soaking time. [Sr] in leached subsamples (open circles in Fig 1) were also similar to each other except for the set of “60°C (2 d),” which contained much lower [Sr], indicating that warmer condition enhances the mobility of Sr in hair.

While the relatively small variation of [Sr] in soaked hair subsamples (solid circles in Fig 1) suggested little exchange of Sr between hair and ambient water, the change of ^87^Sr/^86^Sr ratios in those subsamples (solid squares in Fig 1) clearly showed that Sr in hair was gradually replaced by the Sr contained in the ambient water over time. ^87^Sr/^86^Sr in soaked hair changed from the original value (~0.709) to the water value (~0.714) after 16 days soaking for both mid hair and tip hair subsamples. Even with only 10 minutes of soaking, ^87^Sr/^86^Sr of the hair changed to be significantly different (0.7109 and 0.7107 for mid and tip hair, respectively) from the original values of ~0.709 (Fig 1 and Table 4). Such a fast exchange of Sr between hair and water implies that hair can easily be contaminated during contact with ambient water when the Sr concentration is high enough. For comparison, [Sr] is generally less than 1 μg/kg in snow [22], less than 1 μg/L in precipitation [23], variable from ~10 to ~10,000 μg/L in rivers [24], and approximately ~8000 μg/L in seawater [25].

### Exogenous Sr in soaked hair

We calculated the fraction of exogenous Sr in each subsample using the binary mixture model. We defined the fraction of exogenous Sr in hair as *f* and the fraction of original Sr in hair as 1−*f*. A binary mixture model gives:

$$f\cdot {\left(\frac{{}^{87}Sr}{{}^{86}Sr}\right)}_{exogenous}+\left(1-f\right)\cdot {\left(\frac{{}^{87}Sr}{{}^{86}Sr}\right)}_{original}={\left(\frac{{}^{87}Sr}{{}^{86}Sr}\right)}_{measured}$$

**Fig 2:**
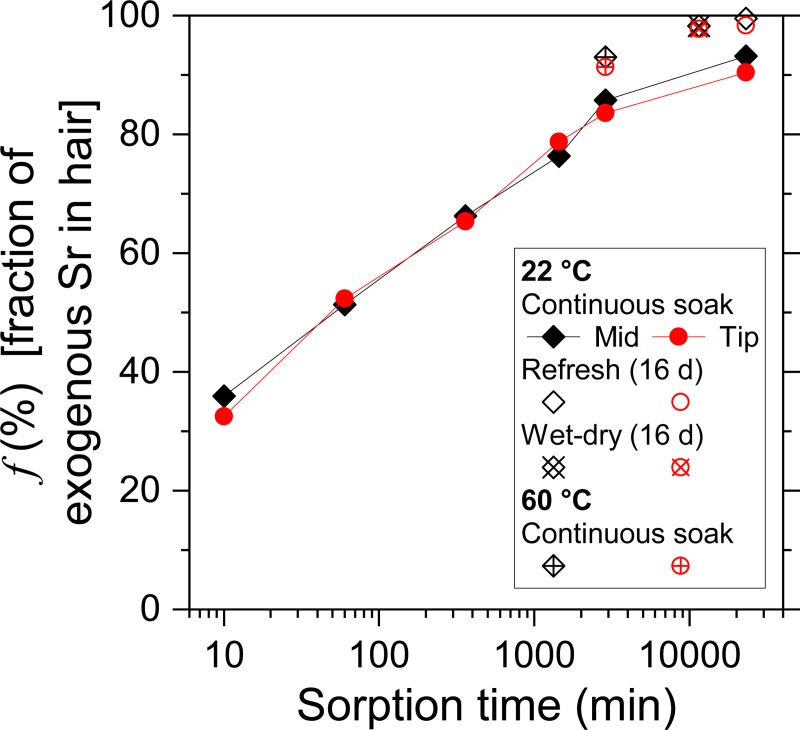
Fraction of exogenous Sr change over time in soaked human hair.

So

$$f=\frac{{\left(\frac{{}^{87}Sr}{{}^{86}Sr}\right)}_{measured}-{\left(\frac{{}^{87}Sr}{{}^{86}Sr}\right)}_{original}}{{\left(\frac{{}^{87}Sr}{{}^{86}Sr}\right)}_{exogenous}-{\left(\frac{{}^{87}Sr}{{}^{86}Sr}\right)}_{original}}$$

where the (^87^Sr/^86^Sr)_original_ represents the original ^87^Sr/^86^Sr ratio in hair before soaking, (^87^Sr/^86^Sr)_exogenous_ represents the ^87^Sr/^86^Sr ratio of the river water, and (^87^Sr/^86^Sr)_measured_ represents the measured ^87^Sr/^86^Sr of each hair sample. The calculated fractions of exogenous Sr (*f*) in all samples are reported in Table 4, and the *f* of soaked subsamples are shown in Fig 2.

At 22°C, the fraction of exogenous Sr incorporated in the soaked hair subsamples increased from 0% to ~30% in only 10 minutes and to ~90% in 16 days, following a logarithmic relationship (Fig 2). Considering that the [Sr] did not increase significantly in the samples with longer soaking time (Fig 1), the dramatic increase of the fraction of exogenous Sr in hair indicated that hair not only sorbed the external Sr from ambient water but also released the internal Sr into ambient water. The fast exchange of Sr between the hair and the ambient water suggests a bidirectional process.

Fig 2 also shows that the fraction of exogenous Sr in the subsamples soaked for 2 days at a warmer temperature (60°C) was similar to the subsamples kept at 22°C for 16 days, indicating the warmer temperature accelerated the exchange of Sr by approximately eight times. The replacement of water with a wet-dry cycle also enhanced the Sr exchange and resulted in nearly complete replacement (~98%) of the Sr contained in hair from the original to the exogenous.

The similarity between the mid and tip hair subsamples suggests that the Sr exchange rate with ambient water was likely uniform for the hair from one individual (Fig 2), while the root should be tested in future studies. Nonetheless, the rate and extent of Sr exchange might differ between individuals, with various hair color, thickness, quality (cuticle completeness), etc. They may also differ between different types of hair among various mammalian species. Besides, the ionic strength of water may also affect the rate and extent of Sr exchange (e.g., seawater vs. groundwater vs. river water). Therefore, future studies on different types of hair and water are needed to provide a more comprehensive understanding of the rate and extent of Sr exchange between hair and water. In a broader sense, this technique might be applied to other materials involving keratin (e.g., wool, feature, clothing, textiles) in the future.

### Acid leaching of hair to identify recent travel

The acid leaching method used in this experiment (3 × 10 min 0.1 M HCl under sonication) did not completely remove the exogenous Sr signal from the soaked subsamples as all leached subsamples contained at least 8.9% of residual exogenous Sr (Fig 1 and Table 4). Moreover, the leachates contained not only the exogenous Sr but also the original internal Sr leached out from the hair as much as 55%, resulting in dissimilar ^87^Sr/^86^Sr ratios from the ambient water. Therefore, the acid leaching procedure in this experiment did not recover either the original ^87^Sr/^86^Sr of the hair or the ^87^Sr/^86^Sr of the water (i.e., the exogenous Sr) from the partially contaminated subsamples. Although the leachate of “Refresh (16 d)” and “Wet-dry (16 d)” subsamples provided the ^87^Sr/^86^Sr ratio of the ambient water (0.714), such information could be obtained without the acid leaching process because the Sr in those subsamples had been completed replaced by the exogenous Sr. Because of the rapid and substantial exchange of Sr between hair and water found in this experiment, it would be challenging for any chemical washing method to recover the ^87^Sr/^86^Sr ratios of either the pure endogenous or exogenous Sr in partially contaminated hair samples. However, it may be worth conducting similar sorption and leaching experiments on more hair samples, as the effect of acid leaching on the Sr in hair might differ among them.

Also shown in Fig 1, the difference of the ^87^Sr/^86^Sr ratio between the leached hair (open squares) and its corresponding leachate (open squares with the cross) differed the most for the hair subsamples with the shortest soaking time, 10 min. Such difference can be illustrated quantitatively as

$$\Delta {}^{87}Sr/{}^{86}Sr_{\left(leached-leachate\right)}\equiv \left|{}^{87}Sr/{}^{86}Sr_{\left(leached\;hair\right)}-{}^{87}Sr/{}^{86}Sr_{\left(leachate\right)}\right|$$

and the Δ^87^Sr/^86^Sr_(leached-leachate)_ values of all subsamples were calculated and reported in Table 4. As the soaking time increased from 10 min to 16 days, the Δ^87^Sr/^86^Sr_(leached-leachate)_ value decreased from ~0.002 to <0.0001. The Δ^87^Sr/^86^Sr_(leached-leachate)_ values of the “Refresh (16 d)” and “Wet-dry (16 d)” subsamples were even smaller, indicating the exchange of Sr between those hair subsamples and the water had reached equilibrium. The Δ^87^Sr/^86^Sr_(leached-leachate)_ values of the subsamples in warmer condition (“60°C (2 d)”) were smaller than those at 22°C (“2 d”).

Generally speaking, the original hair subsamples and those with long soaking time showed a small difference of the ^87^Sr/^86^Sr ratio between the leached hair and its corresponding leachate (Δ^87^Sr/^86^Sr_(leached-leachate)_ < 0.00020), while the subsamples with short soaking time showed the much larger difference (Δ^87^Sr/^86^Sr_(leached-leachate)_ > 0.00020). Therefore, Δ^87^Sr/^86^Sr_(leached-leachate)_ may be used as a tool to identify the alteration of Sr in hair due to the change of environmental Sr.

From the result of this experiment, we have recognized that the Sr in our original hair sample was probably not the endogenous Sr incorporated from the individual’s blood in the hair follicle, but the Sr of bath or shower water. The Sr in our original hair sample had reached equilibrium (Δ^87^Sr/^86^Sr_(leached-leachate)_ < 0.00020), and thus can be considered a single endmember in the soaking experiment of this study. Our original hair sample can also be considered an analog of the hair that contains pure endogenous Sr. The calculation of the fraction of (the latest) exogenous Sr using the binary mixing model in the previous section still holds true. On the other hand, for the hair from an individual who changed locations with different ^87^Sr/^86^Sr ratios in tap water in a short period of time, the “original” Sr in the hair may be a mixture of multiple sources (endogenous Sr from diet + exogenous Sr from location 1 + exogenous Sr from location 2 + …). For these cases, the binary mixing model approach presented in the previous section would not be applicable.

### Role of hair Sr isotopic analysis in provenance and forensic studies

Sr isotopic analyses of hair samples have successfully revealed the existence of multiple sources of Sr with variable ^87^Sr/^86^Sr related to the movement of the individual. One modern horse with known travel history showed a longitudinal variation of ^87^Sr/^86^Sr ratio in its tail hair while, in contrast, a stationary horse did not [1]. Modern humans with known travel history also showed longitudinal variations of ^87^Sr/^86^Sr ratio in scalp hair [6,7]. The longitudinal variation of ^87^Sr/^86^Sr ratio in the hair of the over 3000-year-old “Skrydstrup Woman” from Denmark suggested her migration from a region outside of modern Denmark to the Skrydstrup area [9]. However, it can be challenging to retrieve ^87^Sr/^86^Sr of the pure endogenous Sr in hair for the determination of the region of origin, especially in archaeological and forensic settings [26,27].

As shown in Fig 2, with only 10 minutes of soaking, the Sr signal in hair can be altered more than 30% due to the fast exchange of Sr between hair and river water. It raises a concern about the potential alteration of the ^87^Sr/^86^Sr ratio of the hair which has contacted water. Temperature also accelerates the exchange of Sr between hair and water as much as eight times (Fig 2). Since modern humans take showers, baths, or both at warm temperatures regularly, the Sr isotope signal in hair can hardly be unaltered from the ^87^Sr/^86^Sr ratio resulting from the diet. Such concern has been noticed in a study of human hairs where the authors suggested that bathing with tap water may be an important contributor of Sr to hair [28,29], as well in a study of human fingernails where the authors claimed that, despite ^87^Sr/^86^Sr of fingernail clippings suggested multiple location signals, those signals were likely incorporated through bathing water [30]. The related study on pig tissues found that ^87^Sr/^86^Sr ratios in bristle samples were distinctly different from the ^87^Sr/^86^Sr ratios of other internal tissues, indicating the existence of exogenous Sr in the bristle [16].

Therefore, in provenance studies, caution should be exercised with the Sr isotopic analyses of hair samples from unknown regions of origin due to the possibility that the hair ^87^Sr/^86^Sr ratios have been altered by the Sr exchange with the ambient water on site. When ^87^Sr/^86^Sr in hair is proposed to determine the region of origin of unidentified individuals, the efficacy of the method should be independently evaluated. Here are a few examples where ^87^Sr/^86^Sr ratio in hair may be safely used as a provenance tool: (1) if the hair sample rarely gets in contact with any water, such as giraffe tail hair [19] and archaeological hair samples isolated from water; (2) if the hair sample only contacts the water which contains little Sr such as snowmelt; or (3) if the individual is stationary and the endogenous and exogenous Sr contain the same ^87^Sr/^86^Sr ratio.

Although the direct application of hair Sr isotopic analysis in provenance studies may be limited, it can be used in forensic studies to provide a quantitative estimation of the water contact time. As shown before, the correlation between the fraction of exogenous Sr and the soaking time of hair sample can be established based on the known parameters such as the final hair ^87^Sr/^86^Sr ratio, the original hair ^87^Sr/^86^Sr ratio, the original water ^87^Sr/^86^Sr ratio, and the temperature record. Fig 2 shows the change of the fraction exogenous Sr in soaked hair over time, which quantitatively demonstrates the rate and extent of Sr exchange between the hair and the ambient water. In such cases, Sr isotopic analysis can be applied to estimate the length of soaking time. For example, the travel time of an individual with known region of origin may be estimated if the ^87^Sr/^86^Sr ratios of the original hair and the local tap water and the information of the frequency, length, and temperature of showering/bathing can be obtained; the exposure time of a dead body in a water pool may be estimated if the ^87^Sr/^86^Sr ratios of the original unsoaked hair and the water in the pool, and the recent temperature record are known.

In some situations, the correlation between the fraction of exogenous Sr and the soaking time of hair samples cannot be established because, for example, the original hair ^87^Sr/^86^Sr is not available. Consequently, it is not possible to use the Sr isotopic analysis to estimate soaking time in those situations. However, the combination of acid leaching and Sr isotopic analysis can identify recent alteration of Sr in hair by checking the Δ^87^Sr/^86^Sr_(leached-leachate)_. As shown in the previous section, the difference of ^87^Sr/^86^Sr ratio between a leached hair sample and its leachate, Δ^87^Sr/^86^Sr_(leached-leachate)_, can be used as a tool to identify the alteration of Sr in hair due to the change of environmental Sr, which may indicate a recent movement of the individual to a new locality with a different ^87^Sr/^86^Sr or a tap water change due to management practices, i.e. municipalities change their waters sources. Meanwhile, the value for Δ^87^Sr/^86^Sr_(leached-leachate)_ used as a threshold to identify travelers can differ and should be evaluated carefully because ^87^Sr/^86^Sr of hair and water vary case by case. For instance, Tipple et al. [29] selected Δ^87^Sr/^86^Sr_(leached-leachate)_ > 0.00036 as the criteria to identify recent travel of a subject, while Δ^87^Sr/^86^Sr_(leached-leachate)_ > 0.00020 is appropriate to identify the short water contact in this study.

## Conclusion

When human hair was soaked in river water, a fast, bidirectional exchange of Sr happens between the hair and the water. More than 30% of the endogenous Sr in the hair sample was replaced by the exogenous Sr from the ambient water for as short as 10 minutes at room temperature (22°C). After 16 days of soaking, the Sr in hair was almost entirely replaced by the Sr from water. Warmer temperature (60°C) accelerated the exchange of Sr as much as eight times compared to room temperature. Wet-dry cycles enhanced the Sr concentration in hair after 16 days and increased the fraction of exogenous Sr.

The acid leaching process did not remove all exogenous signal from hair. At the same time, it produced leachate with a mixture of both endogenous and exogenous Sr. Therefore, the acid leaching used in this study could not recover the ^87^Sr/^86^Sr ratio of either the pure exogenous or endogenous fractions in partially contaminated hair samples.

Based on the result of this study, we suggest that care should be applied with the Sr isotopic analysis of hair samples to determine the region of origin of unidentified individuals. On the other hand, if the correlation between the fraction of exogenous Sr and the soaking time of the hair sample could be established, Sr isotopic analysis of hair may be used in forensic studies to estimate the length of water contact time. If such correlation cannot be established, the combination of acid leaching and Sr isotopic analysis of hair samples may still be used to identify recent changes in exogenous Sr pool, including travels and changes in water sources, by checking the difference of ^87^Sr/^86^Sr ratio between a leached hair sample and its leachate, Δ^87^Sr/^86^Sr_(leached-leachate)_, although the criteria should be selected carefully for each study.
